# Biorefinery methods for extraction of oil and protein from rubber seed

**DOI:** 10.1186/s40643-021-00386-2

**Published:** 2021-06-08

**Authors:** Miao Yang, Wenlei Zhu, Hui Cao

**Affiliations:** Beijing Key Lab of Bioprocess, College of Life Science and Technology, Beijing University of Chemical Technology, Beijing, 100029 China

**Keywords:** Rubber seed kernels, Screw press expelling, Solvent extraction, Protein extraction

## Abstract

Rubber seeds are a by-product of rubber production and are rich in oil and protein. Upgrading of rubber seeds to produce proteins, oils and feedstock can generate additional revenue for rubber production and reduce waste. The present study investigates the effects of different pre-treatments and extraction methods to determine the optimal methods to produce oil and protein from rubber seed kernels. Mechanical expulsion using a screw press and solvent extraction using n-hexane were employed for oil separation. The highest oil recovery efficiency of 95.12% was obtained using rubber seed meal that was pre-dried at 105 ℃. The sequential water–alkaline treatment was ideal for achieving high protein recovery while reducing the protein denaturation that can result from high operating temperatures and organic solvent contact. Over 90% of the total protein from rubber seed kernels could be recovered. Separating oil from kernels using hexane followed by protein extraction from the meals by enzymatic treatment provides a suitable method for comprehensive utilization of rubber seeds. 
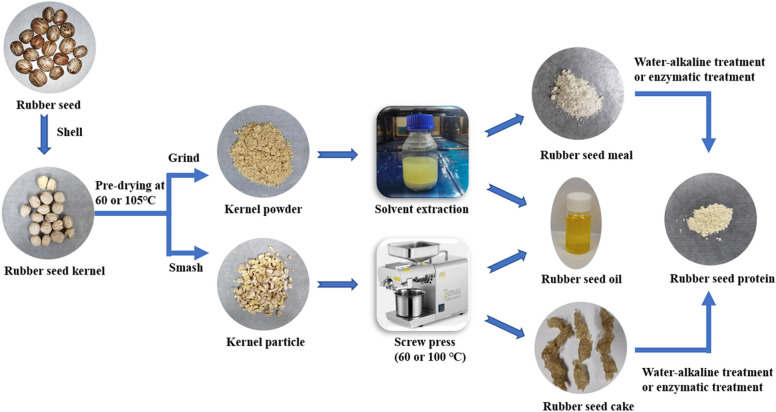

## Introduction

Rubber seeds are an abundant by-product in rubber plantations, with annual production between 136–2000 kg/hectare (Zhu et al. 2014); however, only 25% of rubber seeds are used for seeding and the residual 75% are wasted (Indonesian Directorate Generale of Plantation 2010). Therefore, the full utilisation of rubber seeds can provide a significant source of additional revenue from rubber production. This study focused on finding the optimal methods to produce oil and protein from rubber seed kernels.

The dry matter of the kernel contains 40–50% oil, which is present in the highest amount (Ikwuagwu et al. 2000). Rubber seed oil is an potential product that is currently getting more attention as an alternative feedstock for biodiesel production (Sai Bharadwaj et al. 2019; Samart et al. 2019). However, valorisation of oil alone may not be economically feasible. Separation and use of all fractions to get a better value are envisaged. Rubber seed cakes from oil separation contains 20–28% protein (Widyarani et al. 2017). Oil separation followed by protein extraction from cakes is the optimal process to obtain both oil and protein from rubber seed.

Mechanical pressing and solvent extraction are two common methods to separate oil from oil seeds. Mechanical pressing includes hydraulic pressing and screw pressing, both of which are suitable for separating oil from kernel seeds (Gao et al. 2018; Santoso et al. 2014). Compared to hydraulic pressing, screw pressing is a kind of continuous mechanical pressing, and the pressing temperature is controllable (Pighinelli and Gambetta 2012). Lee et al. (2013) demonstrated that supercritical carbon dioxide extraction can be used to separate rubber seed oil, but the equipment is expensive and the oil recovery is relatively low, so it is not suitable for industrial production. Studies on rubber seed protein extraction are limited. Widyarani et al. (2014) reported a maximum protein recovery of 71% obtained by alkaline extraction and assessed a one-step combined oil and protein extraction using an aqueous enzymatic method, which produced a protein recovery of 67% and a lower oil recovery of 34%. Alkaline and enzymatic extraction methods have been previously investigated to extract protein from agricultural residues such as brewer’s spent grain, rapeseed press cakes and rice bran (Forssell et al. 2008; Niemi et al. 2013; Phongthai et al. 2016; Rommi et al. 2015).

To achieve the most efficient use of rubber by-products, the present study aims to simultaneously obtain high oil and protein recovery from rubber seeds. The effects of different pre-treatments and extraction methods on oil and protein recovery were investigated. Screw press and solvent extraction are investigated for oil separation. Sequential alkaline and enzymatic treatments were used for protein extraction. The method suitable for rubber seed utilization was determined by comprehensive consideration of oil recovery and protein recovery. Additionally, this study gives a prospect of the application of rubber seed oil, protein and residues.

## Materials and methods

### Materials and chemicals

Fresh rubber seeds were kindly provided by Huakun Biotechnology Company, located in the Yunnan province of China. Fresh seeds stored in open containers at room temperature until use.

The chemicals used in the experiments and analysis were of analytical grade. Alkaline protease (S10154) was purchased from Shanghai Yuanye Biotechnology Co. Ltd and stored at 4 ℃ until use. As specified by the manufacturer, the activity of the protease was 200 U/mg, the temperature range was 20–60 ℃, and the pH range was 9–11.

### Pre-treatment

To prepare the rubber seeds for oil and protein extraction, all seeds were mechanically de-hulled and the kernels were separated. The rubber seed kernels were cut into small pieces to facilitate drying. Kernels were dried at either 60 ℃ for 6 h or at 105 ℃ for 3 h to reduce the water content from 29% to 4–5%. Flaxseeds were prepared and dried under the same conditions for subsequent comparison.

### Oil separation

Pre-dried kernels of different sizes were subjected to screw pressing or solvent extraction using n-hexane.

#### Oil recovery by screw pressing

Batches of pre-dried rubber seed kernels were crushed to different degrees and passed through 12.5, 8.0, 6.0, 5.0, 4.0, 3.0, 2.0 and 1.6 mm sieves, respectively. Three batches of kernels with average particle diameters of 3.5, 7.0, and 10.25 mm were selected and pressed at 100 ℃. Pressing was performed using a small-scale commercial screw press that operates at temperatures between 60 ℃ and 280 ℃ with a maximum input of 4 kg/h. The applied experimental temperatures were 60 ℃ and 100 ℃ and the input rate was 4 kg/h. Pressing was performed in duplicate for each condition, using 150 g of kernels per batch. Press cakes were stored at 4 ℃ for further use. Pre-dried flaxseeds were also subjected to screw pressing under the same experimental conditions.

#### Oil recovery by solvent extraction

The pre-dried rubber seed kernels and flaxseeds were ground to ≤ 0.5 mm. To achieve a 1:7 solid-to-solvent ratio, 10 g feedstock was suspended in 70 ml n-hexane in GL 45 bottles. The flasks were placed in a water bath at 60 ℃ and shaken at 220 rpm for 2 h. The remaining solid was recovered by centrifugation (6000*g*, 10 min) and the liquid phase was stored for further analysis. The solid material was then submitted to the same extraction process with fresh n-hexane two more times. After the third extraction, the oil-rich extracts were combined and mixed, before hexane evaporation and weighing of the oil. The resulting meal by-product from the solvent extraction was dried at 60 ℃ to remove residual hexane and stored at 4 ℃ for further use. The experiments were performed in duplicate.

### Protein extraction

#### Protein extraction by water–alkaline–alkaline treatment

For the sequential treatment, de-fatted materials were first mixed with de-ionised water (1:15 solid-to-liquid ratio) and incubated at 25 ℃ and shaken at 220 rpm for 1 h. The liquid and solid fractions were separated by centrifugation (5000*g*, 10 min) and the liquid phase was stored for analysis. After treatment, the residual solids were subjected to twin sequential 1 h alkaline extractions using 0.1 M NaOH (1:10 solid-to-liquid ratio) at 25 ℃ and 220 rpm. Both extracts were collected by centrifugation (5000*g*, 10 min) for further analysis. All the above steps were also performed at 60 °C. The experiments were performed in duplicate.

#### Enzymatic protein extraction

Enzymatic treatment was performed at 25 ℃ and 60 ℃. The de-fatted feedstock was mixed within the reaction system (de-ionised water, pH 9, 1:15 solid-to-liquid ratio). Alkaline protease (3.5% v/w) was added to the mixture before extraction. The reactions were performed in GL 45 bottles and kept in a shaker incubator at 220 rpm, for 1 or 6 h. The system was heated to 98 ℃ for 10 min to stop the reaction. The liquid fraction was collected by vacuum filtration for further analysis. The experiments were carried out in duplicate.

### Analytical methods

#### Ash and moisture contents

The ash content was calculated from the weight difference before and after heating the products in a muffle furnace at 600 ℃ until reaching a constant weight. The moisture content was determined from the weight difference before and after oven drying at 105 ℃ until reaching a constant weight.

#### Starch, cellulose, hemicellulose and lignin contents

The starch content was determined using a starch assay kit (Solarbio Starch Content Assay Kit), according to the manufacturer’s instructions.

Cellulose, hemicellulose and lignin contents were determined following the NREL standard protocols (Sluiter et al. 2004). Press cakes were mixed with 72% (w/w) H_2_SO_4_ and incubated at 30 ℃ for 1 h. Each sample was diluted to 4% (w/w) H_2_SO_4_ by adding de-ionised water before incubation at 120 ℃ for 1 h. The hydrolysate was cooled to room temperature and the supernatant liquid fraction was filtered through a 0.22 μm syringe filter. High-performance liquid chromatography (HPLC) was used to determine the concentration of glucose and xylose. The cellulose content was calculated from the concentration of glucose and the hemicellulose content was calculated from the concentration of f xylose. The remaining liquid fraction was filtered using a sand core funnel and then the funnel was dried at 105 ℃ for 24 h. The lignin content was determined from the weight difference before and after drying.

#### Oil content

The pre-dried rubber seed kernels and flaxseeds were ground using a grinder, and then a 10 g sample of each was used to determine the oil content. The oil contents of the rubber seed kernels, flaxseeds and press cakes were analysed using Soxhlet with n-hexane as the extracting solvent at 70 ℃ for 6 h. The extraction were repeated using fresh hexane until the extracts became colorless. The extracts were subjected to hexane evaporation, before weighing.

#### Fatty acid composition

Oil was prepared from seeds that were pre-dried at 60 ℃. The fatty acids were methylated before the composition was determined using gas chromatography–mass spectrometer (GC–MS). The GC–MS was equipped with a PEG-20 M, column and the analyses were conducted under a 280 ℃ input temperature, 50 ℃ column temperature, and a two-stage temperature cycle programmed as follows: 50 ℃ for 50 min, increased to 120 ℃ at 5 ℃/min, 120 ℃ for 2 min, finally increased to 280 ℃ at 10 ℃/min, and finally held at 280 ℃ for 2 min.

#### Biodiesel fuel properties

The fuel properties of rubber seed oil (density, kinematic viscosity, oxidative stability, iodine value, cloud point, pour point, high heating value) were estimated using “the BiodieselAnalyzer^©^ Ver. 3.3” (Talebi et al. 2014) based on the fatty acid compositions of rubber seed oil.

#### Solid and moisture content analysis of screw-pressed rubber seed oil

After centrifugation, the solid matter was separated from the oil obtained by screw pressing, washed with n-hexane, dried in a vacuum and weighed. The water content was measured using a Karl Fischer Moisture Analyzer (Mettler C20SD). After blank titration, drift determination and reagent calibration, the water content of the samples was determined.

#### Protein determination

The protein content of the rubber seed kernel, extracts and press cakes were analysed by the Kjeldahl method. (Widyarani et al. 2014).

#### Amino acid composition

The extracted protein amino acid composition was analysed using an automatic amino acid analyser (Hitachi L-8900). Samples were hydrolysed with 6 M HCl at 110 ℃ for 24 h. The hydrolysates were dissolved in 0.02 M HCl and loaded into the analyser. Citrate buffer was used at a buffer flow rate of 0.4 ml/min. The column temperature was set to 55 ℃, and the reaction temperature was 135 ℃. Detection was conducted at 440 nm and 570 nm.

#### Calculations

The oil and protein contents were both calculated as the percentage of dry matter. The oil recovery was calculated as the weight of recovered oil divided by the weight of oil in the kernel. The oil yield of the solvent extraction was calculated as the weight of the separated oil divided by the total weight of the feedstock. The protein recovery was calculated as the weight of the separated protein divided by the weight of protein in the kernel. The solid content was calculated as the weight of the solid matter divided by the weight of the separated oil. The amount of all amino acids after hydrolysis were summed to calculate the total amino acids.

## Results and discussion

### Rubber seed composition

Rubber seeds consist of two parts: the kernel and the shell. Rubber seeds have a relatively high moisture content (29.92%), most of which is stored in the kernel (Table 1). The combination of a hard and compact shell and a kernel with high moisture content makes fresh rubber seeds, stored at room temperature prone to mould growth, which can prevent the utilisation of rubber seed resources. Therefore, the moisture content must be substantially lowered (≤ 7%) before rubber seeds can be safely stored (Ebewele et al. 2010). Rubber seeds are also rich in oil; the oil content in dried kernels is 48.78% (Table 1), which is high compared to seeds such as flax, sunflower, and rapeseed (Abitogun et al. 2012; Li et al. 2014).

**Table 1:** Composition of rubber seed, as percentages, based on dry weight (dw) or wet weight (w)

Sample	Unit	Amount
Whole seed		
Weight (fresh)	g	4.7 ± 0.3
Kernel	%-w	58.84 ± 0.71
Shell fraction	%-w	41.16 ± 0.71
Kernel		
Moisture (fresh)	%-w	29.22 ± 1.03
Oil content	%-dw	48.78 ± 1.15
Protein content	%-dw	19.12 ± 1.12

As shown in Table 2, rubber seed cake (RSC) is mainly composed of protein (36.60% w/w) and polysaccharides in the form of starch (15.28% w/w), cellulose (8.45% w/w) and hemicellulose (11.62% w/w).The protein content in RSC is higher than that in other agro-industrial feedstocks (Tessari et al. 2016), making RSC a low-cost alternative protein source that can be used for further industrial applications.

**Table 2:** Composition of rubber seed cake (RSC)

Component	Composition of RSC (g/100 g)
Protein	36.60 ± 1.70
Starch	15.28 ± 0.04
Cellulose	8.45 ± 0.53
Hemicellulose	11.62 ± 0.42
Lignin	4.29 ± 0.84
Lipids	8.63 ± 0.28
Moisture	6.21 ± 0.09
Ash	5.49 ± 0.03
Other mass	3.43

### Oil recovery

#### The effect of particle size on oil recovery

**Fig. 1:**
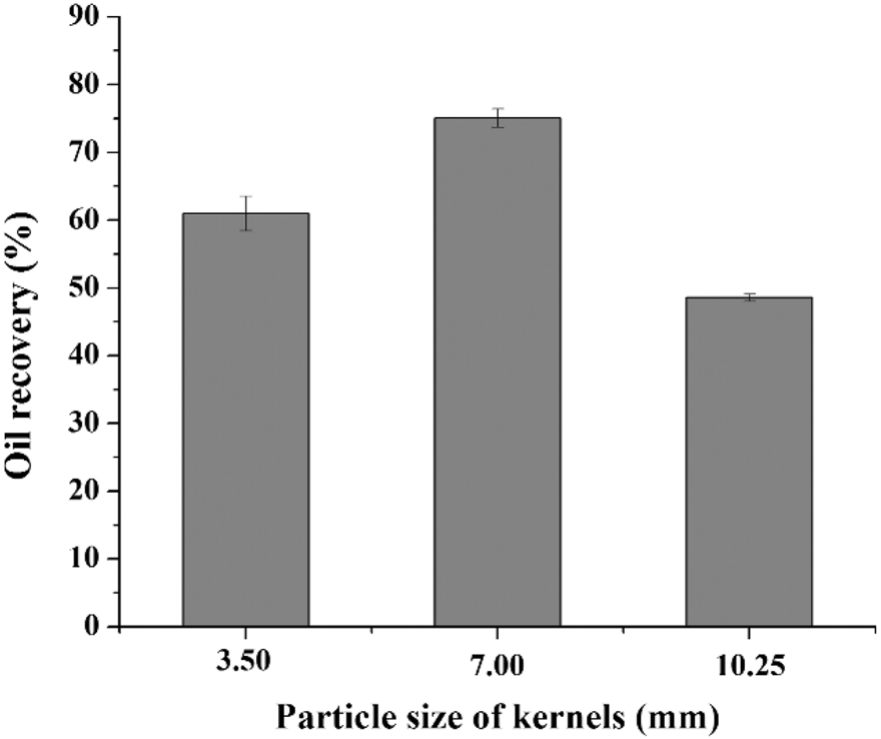
Oil recovery from rubber seed kernels of different particle sizes

**Fig. 2:**
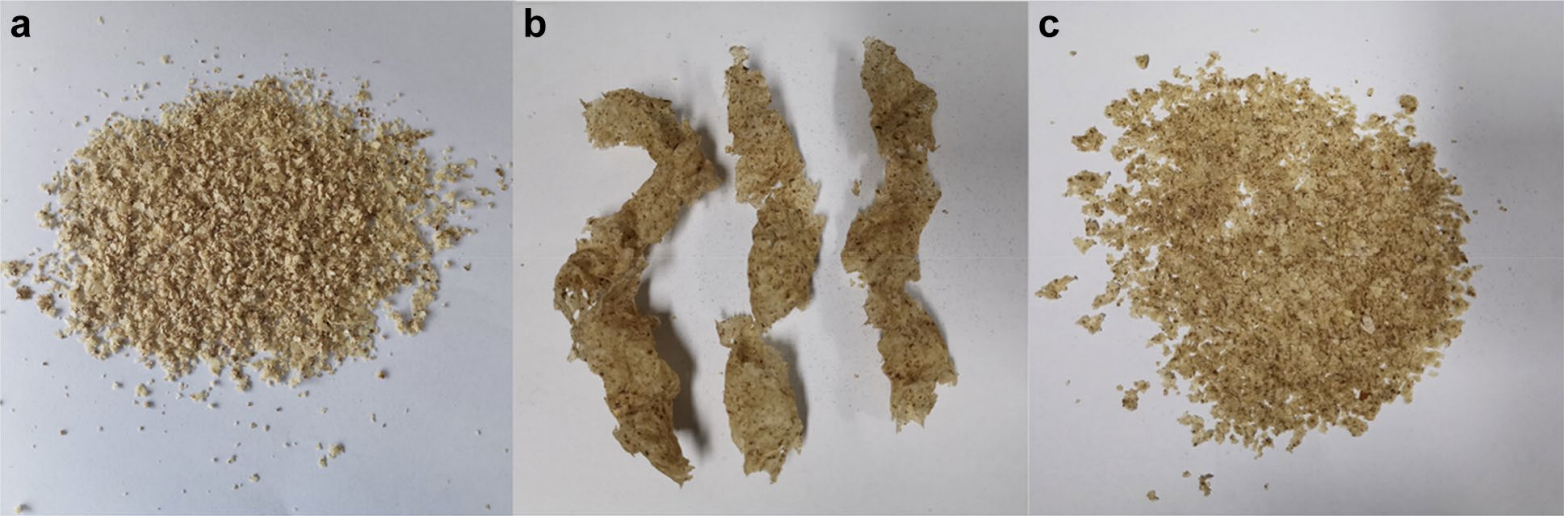
Cake obtained by pressing oil from rubber seed kernels with different particle sizes (**a**) 3.5 mm; (**b**) 7 mm; (**c**) 10.25 mm

The particle size of kernels influences the percentage of oil recovery from screw pressing (Fig. 1). The optimum kernel size for oil extraction by screw pressing is 7 mm. In addition, the shape of the cake obtained by pressing rubber seed kernels varied among different particle diameters, as shown in Fig. 2. The cake obtained by pressing 7 mm kernels is continuous and complete. The cake becomes fragmented when the particle size is too large or too small, which affects the discharge. Therefore, kernels with a particle size of 7 mm are selected for screw pressing.

#### The effect of oil separation method on oil recovery

**Fig. 3:**
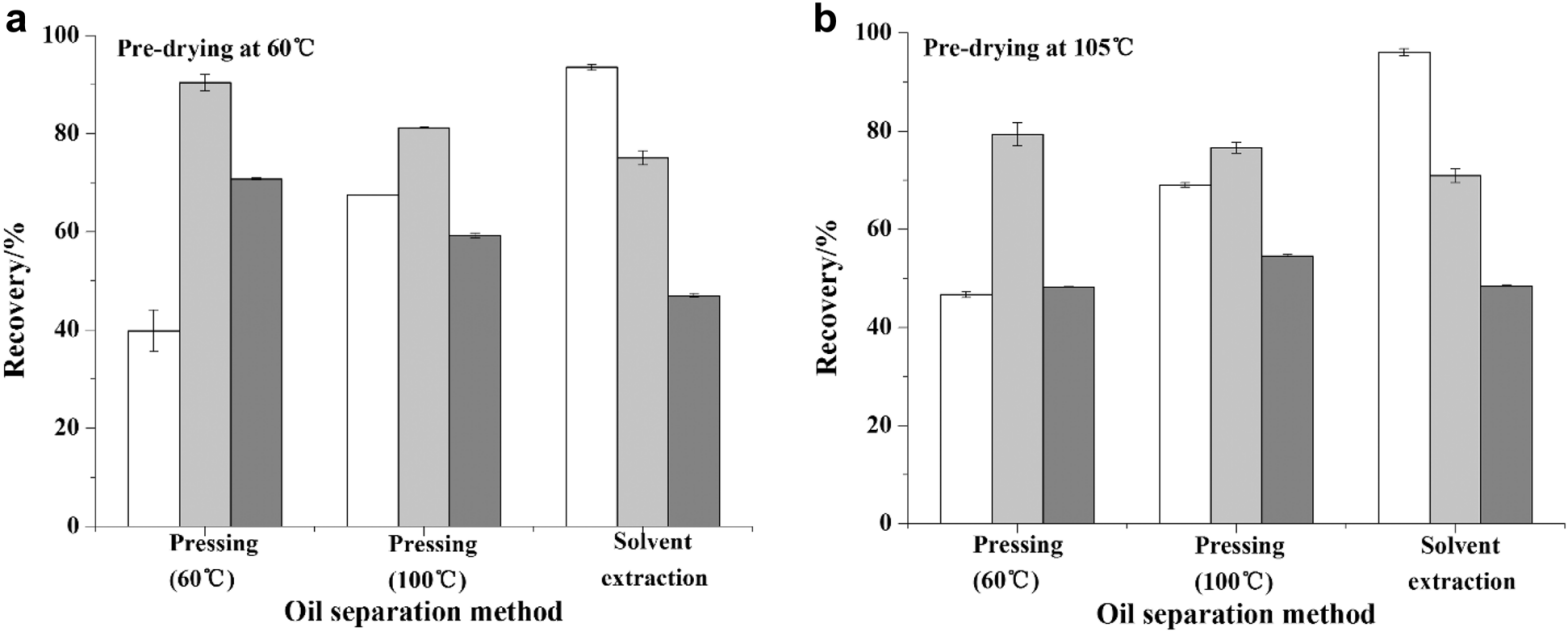
Oil recovery () and protein recovery from water–alkaline–alkaline treatment () and from enzymatic treatment ()

We compared the oil recovery between a screw press and solvent extraction method for oil separation. Mechanical screw pressing seldom exceeds an efficiency of 90% but provides the advantage of producing products without added chemicals. Solvent extraction typically achieves a higher oil recovery rate than mechanical extraction (Santoso et al. 2014). In line with previous findings, the experimental results show that the oil recovery rate from screw pressing was much lower than that obtained using solvent extraction (Fig. 3a, b). As shown in Fig. 3a, b, the highest oil recovery achieved using the screw press was 69.01%, which was obtained from rubber seed kernels pre-dried at 105 ℃ and pressed at 100 ℃. The oil recovery increased by 22.78–27.64% when the pressing temperature was increased from 60 to 100 ℃, due to reduced percolation resistance resulting from the lower viscosity of the oil at higher temperature. This temperature effect was not observed when using a hydraulic press (Santoso et al. 2014).

In our study, we used fresh hexane to repeat the extraction process three times to simulate the countercurrent extraction process used in industrial production. Solvent extraction gives the highest oil recovery of 95.12%, using rubber seed kernels pre-dried at 105 ℃, which was 26.11% higher than that of screw pressing.

#### The effect of pre-drying temperature on oil recovery

**Fig. 4:**
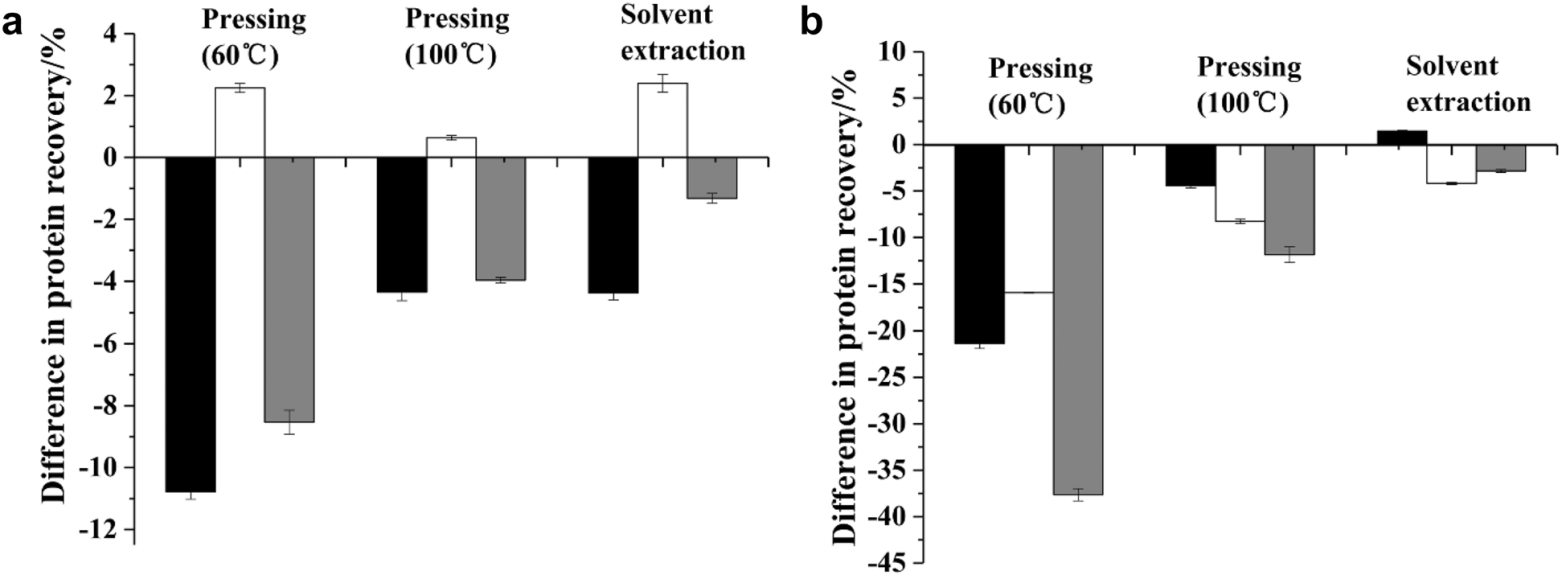
Differences in protein recovery from increasing the pre-dried temperature from 60 ℃ to 105 ℃ () and from increasing the protein extraction temperature from 25 to 60 ℃ (), grey bars () indicate the net influence. **a** protein extraction by water–alkaline–alkaline treatment; **b** Protein extraction by enzymatic treatment

Fresh rubber seed kernels have a relatively high moisture content. Pre-drying is an important step before oil separation, because moisture content influences the oil recovery efficiency achieved by pressing or solvent extraction (Baümler et al. 2010; Martínez et al. 2008). If the kernel moisture content is too low (≤ 2%), the oil cannot be continuously produced during the pressing process and only a paste of oil and cakes will be obtained. In this study, the moisture content of the kernels was 4–5% after pre-drying at 60 ℃ or 105 ℃. More efficient oil recovery was achieved after pre-drying at a higher temperature for all oil separation methods (Fig. 4a, b). After the pre-drying temperature was increased from 60 to 105 °C, the oil recovery rate from screw press increased by 1.54–7.79%, and the oil recovery rate from solvent extraction increased by 2.66%. High-temperature pre-drying disrupts the cells and protein associated with the oil resulting in increased oil expulsion from the kernels.

### Protein recovery

#### The effect of oil separation method on protein recovery

Almost all of the protein in the kernels was retained in the press cakes and meals after the oil separation process. The protein recovery obtained under most experimental conditions is higher than 50%, which is comparable to the protein recovery rates for other agricultural feedstocks such as rice bran (Phongthai et al. 2016), rapeseed press cake (Rommi et al. 2015) and canola meal (Gerzhova et al. 2015).

Overall, protein recovery from press cakes was more efficient than that from meals. During solvent extraction processing, meal particles become drier and more compact, which results in a lower protein recovery rate (Lestari et al. 2011). Another disadvantage of solvent extraction is that the meals produced are powdery. Compared with the flaky cakes generated from pressing, the solid residue of the meal left after oil extraction is more difficult to separate. Our results also demonstrate that the residual oil content of the cakes and meals has little impact on the protein recovery rate. The highest oil recovery was obtained by solvent extraction; however, the protein recovery from the resulting meal is relatively low compared with that of the cakes.

As shown in Fig. 3a, the protein recovery from pressing at 60 ℃ was 9.12–11.53% higher than that from pressing at 100 ℃. This result was attributed to increased protein denaturation at higher temperature. Obviously, the effect of pressing temperature on protein recovery was less than that observed in the solvent extraction.

#### The effect of pre-drying temperature on protein recovery

The protein recovery from press cakes and meals decreased when the kernels were pre-dried at 105 ℃ compared to 60. The protein recovery rate from cakes obtained by screw pressing decreased by 4.61–22.6%, and the protein recovery rate from meals obtained by the solvent extraction decreased by 5.09%. Our findings were consistent with a previous study that reported a higher protein yield from non-heated rapeseed press cakes compared with heated rapeseed meals (Tan et al. 2011). Östbring et al. (2020) also observed a reduction in protein yield after cold-pressed rapeseed cakes were exposed to heat (80 ℃ for a few seconds). Our hypothesis for the observed decrease in protein recovery is that a higher temperature partly denatures the protein, resulting in protein coagulation and decreased solubility.

The influences of increasing the pre-drying temperature and protein extraction temperature on the protein recovery rate are presented in Fig. 4a, b. Protein extractability is controlled by diffusion (Russell and Tsao 1982) and diffusivity increases when the protein extraction temperature is increased; hence, the protein recovery was higher when the protein was extracted by water–alkalin–alkaline treatment (Fig. 4a). The effect of the pre-drying temperature on protein recovery was more evident (Fig. 4). The net influence shows a decrease in protein recovery, which suggests that protein coagulation inhibits diffusion even at a higher extraction temperature. Alternatively, protein recovery from enzymatic treatment decreased with higher extraction temperature due to reduced enzyme activity. Overall, the net influence of increasing the pre-drying and extraction temperatures results in a decrease in protein recovery, regardless of the extraction method.

#### The effect of protein extraction method on protein recovery

One major problem that limits protein extraction is the decrease in protein solubility that results from higher operating temperatures or contact with an organic solvent. It has been demonstrated that improved protein solubility can be obtained by increasing the pH in the extraction phase; a pH of 12 yielded the highest protein recovery rate (Aider and Barbana, 2011; Fetzer et al. 2018; Wijesundera et al. 2013). In our study, sequential strong alkaline extraction (0.1 M NaOH) was used to improve protein recovery. We compared the results with those obtained using an enzymatic treatment.

**Fig. 5:**
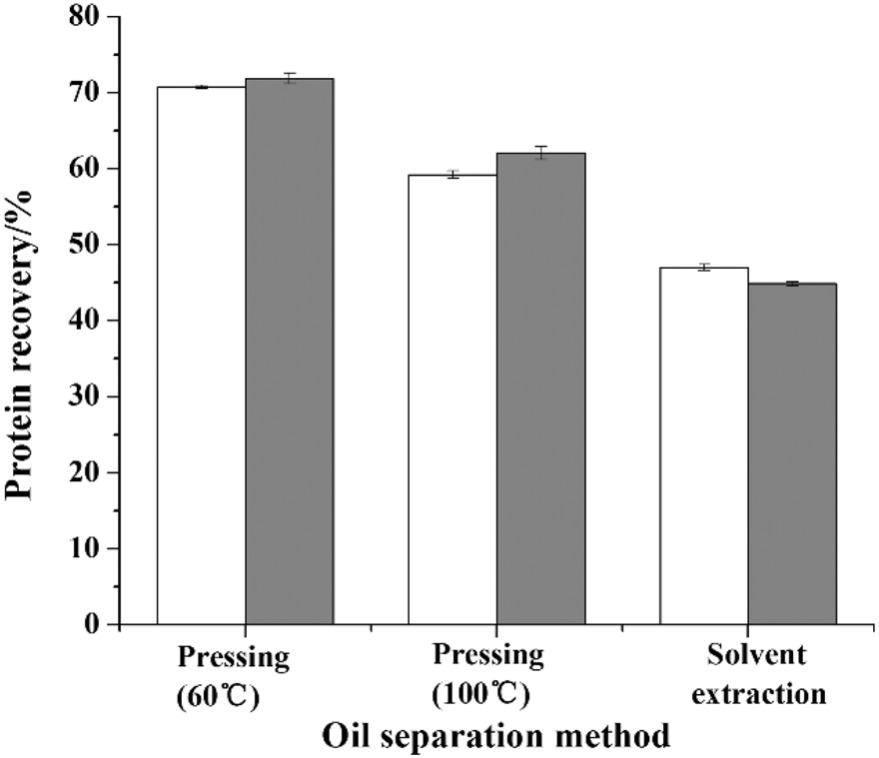
Effect of extraction time on protein recovery. () Protein extractions performed at 25 ℃ for 1 h; () Protein extractions performed at 25 ℃ for 6 h

The highest protein recovery (90.37%) was obtained from the water–alkaline–alkaline treatment using cakes produced by pressing at 60 ℃ of kernels pre-dried at 60 ℃. Using cakes obtained under the same experiment conditions, the highest protein extraction rate obtained by enzymatic treatment was 70.76%. The most popular methods for extracting protein from agro-food residues are enzyme-assisted extraction and alkaline extraction, with reported protein recovery ranging from 13.2to 80%, and 15 to 45.1%, respectively (Contreras et al. 2019). In this study, water–alkaline–alkaline treatment was adopted to obtain a higher protein recovery rate. However, continuous extraction in a strong alkaline environment caused a large amount of polysaccharides in the cakes/meals to be hydrolyzed, which affected the subsequent application of the residues from protein extraction, and the large amount of waste water produced in the continuous extraction process is not easy to handle, so the enzymatic treatment is more suitable for industrial production. Our study explores the effect of enzymatic treatment time on protein recovery rate, as shown in Fig. 5. The results proved that the protein extraction rate did not change much when the enzymolysis time was extended.

### Composition of rubber seed oil

#### Moisture and solid content of rubber seed oil

The moisture content of RSO was less than 0.3% (Tables 3, 4), which meets the required specifications of crude oil moisture content stipulated by the National Soybean Processors Association. There were no significant differences in moisture content among RSO produced at different operating temperatures. However, the moisture content of the RSO obtained by solvent extraction was relatively low.

**Table 3:** Moisture and solid content of rubber seed oil obtained by pressing

Operating temperature (℃)		Moisture content (%)	Solid content (%)
Pre-dried temperature	Pressing temperature		
60	60	0.075 ± 0.003	13.5 ± 1.17
	100	0.067 ± 0.008	9.09 ± 0.04
105	60	0.070 ± 0.005	13.43 ± 0.22
	100	0.079 ± 0.001	7.95 ± 0.05

**Table 4:** Moisture and solid content of rubber seed oil obtained by solvent extraction

Operating temperature (℃)	Moisture content (%)	Solid content (%)
Pre-dried at 60 ℃	0.024 ± 0.004	/
Pre-dried at 105 ℃	0.035 ± 0.003	/

The solid content of RSO obtained by screw pressing is presented in Table 4. Increasing the pressing temperature from 60 to 100 ℃ resulted in a 4.41–5.48% decrease in solid content. These experimental results are as expected, as oil viscosity decreases with increasing temperature. The lowest RSO solid content (7.95%) was obtained from pressing and pre-drying kernels at high temperatures (100 ℃ and 105 ℃, respectively), which was lower compared to the solid content of palm crude oil (9.7%) obtained by screw pressing (Owolarafe et al. 2002). In industrial production process, solid impurities are removed by filtration (Kong et al. 2015).

#### Fatty acid composition of rubber seed oil

The main fatty acid composition and content of rubber seed oil (RSO) are shown in Table 5. There was no significant difference in the fatty acid composition of RSO obtained by solvent extraction and screw pressing. Taking RSO obtained by solvent extraction as an example, the composition was mainly palmitic acid, stearic acid, oleic acid, linoleic acid and linolenic acid. The content of unsaturated fatty acids in RSO is as high as 80.44%, of which monounsaturated fatty acids represent 23.68% and polyunsaturated fatty acids represent 56.76%. Compared to FSO, RSO had a lower linolenic acid content, but a higher linoleic acid content. Overall, the unsaturated fatty acid content of RSO (80.44% w/w) was similar to that of FSO (85.29% w/w). Furthermore, compared to soybean oil and olive oil, RSO has the highest linolenic acid content (Mohd-Setapar et al. 2013).

**Table 5:** Fatty acid composition of rubber seed oil (RSO) and flaxseed oil (FSO)

Fatty acid	Content (%)		
	FSO (solvent extraction)	RSO (solvent extraction)	RSO (pressing at 60 ℃)
Palmitic acid (C16:0)	7.28 ± 0.18	10.77 ± 0.14	9.82 ± 0.11
Stearic acid (C18:0)	6.24 ± 0.04	8.05 ± 0.44	7.67 ± 0.41
Oleic acid (C18:1)	23.77 ± 0.10	23.68 ± 0.19	22.88 ± 0.35
Linoleic acid (C18:2)	15.83 ± 0.02	36.16 ± 0.50	35.11 ± 0.44
Linolenic acid (C18:3)	45.69 ± 0.03	20.60 ± 0.68	22.28 ± 0.13

#### Outlook for application of RSO

At present, RSO has been industrialised as an edible oil on a small scale. In addition, RSO has potential as a raw material for biodiesel production. We used the fatty acid composition to determine assessed whether RSO is suitable for biodiesel. The estimated fuel properties of RSO compared with other biodiesel materials and the requirements specified by the ASTM D6751 and EN14214 standards for biodiesel are shown in Table 6.

**Table 6:** Estimation of the biodiesel fuel properties of rubber seed oil in comparison with other biodiesel materials and the international standards for biodiesel

Fuel properties	Biodiesel from RSO	Biodiesel from SBO^a^	Biodiesel from SFO^b^	Biodiesel from RO^c^	International standards	
					ASTM D6751	EN14214
Density (g/cm^3^)	0.87	0.88	0.88	0.92	0.82–0.9	0.86–0.9
Kinematic viscosity (mm^2^/s)	3.50	4.22	3.64	4.34	1.9–6	3.5–5
oxidative stability (h)	4.67	4.50	–	–	≧3	≧6
Iodine Value	143.17	–	–	–	ns	≤ 120
degree of unsaturation	151.86	–	–	–	ns	ns
Cloud point (℃)	0.67	–	3.0	–	− 3–15	ns
Pour point (℃)	− 6.09	–	0.00	–	–	–
High heating value (KJ/g)	39.11	–	–	35.60	ns	ns

*SBO* Soybean oil, *SFO* Sunflower seed oil, *RO* Rapeseed oil, ns: not specified

Data are from ^a^ Nogueira et al. (2020), ^b^ Sayed et al. (2020), ^c^ Rezki et al. (2020),

The density of biodiesel can affect viscosity, heating value, fuel performance and air–fuel ratio (Patel et al. 2016). The density of RSO is 0.88 g/cm^3^, which meets the international standard specifications and is similar to that of the biodiesel produced from soybean oil (0.88 g/cm^3^) and sunflower seed oil (0.88 g/cm^3^).

Kinematic viscosity is one of the most important parameters for approving biodiesel as an alternative fuel. High kinematic viscosity leads to poor combustion and large droplet sizes; thus a low kinematic viscosity value is essential. The kinematic viscosity value of RSO is within the specification ranges for all international standards and is comparable to those of biodiesel from soybean oil (4.22 mm^2^/s), sunflower seed oil (3.64 mm^2^/s) and rapeseed oil (4.34 mm^2^/s).

The oxidative stability and iodine value of biodiesels are affected by the degree of unsaturation. High oxidative stability indicates that the biodiesel will have a long shelf life. In addition, a low iodine value is indicative of less susceptibility to oxidation. Due to the high degree of unsaturation, the estimated oxidative stability and iodine value of RSO do not satisfy the limits specified by EN14214 standards. This problem could be solved by mixing RSO with palm oil, which contains lower amounts of unsaturated linolenic acid (Hamidah et al. 2011).

The cloud point is defined as the temperature at which the first wax crystal is formed, and the pour point is the minimum temperature at which fuel flows. For RSO, both parameters are lower than those of sunflower seed oil biodiesel. The pour point is usually at a lower temperature than the cloud point (Knothe and Razon, 2017), which is also true for RSO, as confirmed by our results.

The high heating value is the amount of the heat released by a unit of fuel after complete combustion. The high heating value is not specified by any of the biodiesel standards; however, compared with biodiesel from rapeseed oil, the estimated high heating value of RSO is higher. Overall, the estimated fuel properties of RSO compare favourably to those of biodiesel produced from other oils.

### Composition and application prospect of the extracted protein and residue

The amino acid composition of the protein extracted is shown in Table 7. Acid hydrolysis completely destroyed tryptophan, and glutamine and asparagine were converted to glutamic acid and aspartic acid, thus, these amino acids were absent. The nutritional value of protein used for animal feed is usually determined by the amounts of essential amino acids. The essential amino acids in the extracted protein including threonine, valine, methionine, isoleucine, leucine, phenylalanine and lysine, were present at substantial concentrations. This suggests that rubber seed protein is a suitable substitute for plant protein in animal feed. Babatunde et al. (1990) previously reported that rubber seed kernel protein could be used as a partial replacement for higher quality protein sources in the diet of swine.

**Table 7:** Amino acid composition of protein extracted from rubber seed

Amino acid	Composition (g/100 g)
Threonine	2.25
Valine	4.21
Methionine	1.71
Isoleucine	3.31
Leucine	5.82
Phenylalanine	4.58
Lysine	3.55
Aspartic acid	4.76
Serine	4.35
Glutamic acid	13.11
Proline	4.65
Glycine	6.63
Alanine	4.72
Cysteine	1.49
Tyrosine	3.98
Histidine	1.66
Arginine	6.26
Total	77.04

Nutrients such as protein, starch and oil were also present in the residue from protein extraction. In the sequential water–alkaline treatment, most starch is hydrolysed. As shown in Table 8, the residue from enzymatic treatment is mainly composed of protein (10.51% w/w), starch (21.56% w/w) and cellulose (24.75% w/w). In a previous study, lactic acid fermentation was reported to reduce the fibre content of feedstuffs such as brewer’s spent grain, increasing the protein content (Mladenovi and Djuki 2019), which suggests that lactic acid fermentation could increase the value of the residues as feed. These findings suggest that the residue could be used as a suitable feed.

**Table 8:** Composition of the residue from enzymatic treatment

Component	Composition of residue (g/100 g)
Protein	10.51 ± 1.03
Starch	21.56 ± 0.05
Cellulose	24.75 ± 0.58
Lignin	9.29 ± 0.09
Lipids	4.91 ± 0.34
Moisture	5.32 ± 0.10
Ash	8.87 ± 0.06

## Conclusions

Our study revealed that the efficiency of extracting oil and protein from rubber seed kernels varies significantly depending on the pre-treatment and extraction methods. For screw pressing, high operating temperatures increase the release of oil by breaking the cell structure and lowering the oil viscosity. Proteins associated with the oil are also denatured at high temperatures, which is useful for separating the oil from kernels but negatively impacts protein diffusion. The maximum oil recovery achieved from screw pressing was 69%. The highest oil recovery of 95.12% was achieved by repeated solvent extraction using fresh n-hexane each time. The water–alkaline–alkaline treatment method produced the best protein recovery rate of 90.37% from press cakes; highly alkaline conditions improved the solubility of the denatured kernel protein. However, the disadvantages of sequential alkaline treatment include the production of high volumes of wastewater and loss of starches by hydrolysis, which is not conducive to the subsequent utilisation of residues. Compared with the increase in oil yield, high pre-treatment temperatures had a great impact on protein extraction recovery. Thus, a low pre-treatment temperature is recommended. In general, extracting oil from kernels using hexane followed by protein extraction by enzymatic treatment from the meal provides an approach for comprehensive utilisation of rubber seed kernels.

## Data Availability

All data supporting this article’s conclusion are available.

## Abbreviations

HPLC: High performance liquid chromatography
GC-MS: Gas chromatography-mass spectrometer
RSC: Rubber seed cake
RSO: Rubber seed oil
FSO: Flaxseed oil
SBO: Soybean oil
SFO: Sunflower seed oil
RO: Rapeseed oil
